# Early abortion care during the COVID‐19 public health emergency in Ireland: Implications for law, policy, and service delivery

**DOI:** 10.1002/ijgo.13720

**Published:** 2021-05-18

**Authors:** Alison Spillane, Maeve Taylor, Caitriona Henchion, Róisín Venables, Catherine Conlon

**Affiliations:** ^1^ Irish Family Planning Association Dublin Ireland; ^2^ School of Social Work and Social Policy Trinity College Dublin Dublin Ireland

**Keywords:** abortion, COVID‐19, reproductive health, reproductive rights, telemedicine

## Abstract

Early abortion care became available in Ireland in January 2019. Service delivery involves two consultations with a medical practitioner, separated by a mandatory 3‐day waiting period. The Model of Care for termination of pregnancy initially required in‐person visits. The onset of the COVID‐19 pandemic necessitated significant reductions in in‐person interactions in healthcare. A revised Model of Care for termination of pregnancy, issued for the duration of the pandemic, permits delivery of early abortion care by remote consultation. Significantly, this was introduced without amending the 2018 abortion law. The pandemic precipitated a rapid development in the delivery of abortion care that was not anticipated at the time of abortion law reform only 18 months earlier. We outline the work undertaken to maintain access to abortion care in early pregnancy through the lens of a single community‐level provider and explore what these developments may mean for abortion law, policy, and service delivery.

## Synopsis

COVID‐19 precipitated the introduction of remote consultation for early abortion in Ireland. This paper explores what this may mean for law, policy, and service delivery.

## 1 INTRODUCTION

Abortion in early pregnancy became available in Ireland in January 2019, following decades of campaigning by activists and civil society organizations for the repeal of the country's constitutional abortion ban.^1^ The new law—the Health (Regulation of Termination of Pregnancy) Act 2018—provides for access to abortion on request up to 12 weeks of pregnancy, dated from the last menstrual period (LMP).^2^

Early abortion care initially involved two face‐to‐face visits with a medical practitioner, separated by a 3‐day mandatory waiting period. The Model of Care for early abortion was designed around the provision of early medical abortion (EMA) which involves the use of two medications—mifepristone, followed 24–48 hours later by misoprostol.

EMA up to 9 weeks of pregnancy is provided by community healthcare providers (general practitioners (GPs), and specialist sexual and reproductive healthcare providers, such as the Irish Family Planning Association (IFPA)), through a combination of medical supervision and home self‐management. For pregnancies between 9 and 12 weeks of gestation, patients are referred for hospital‐based abortion care. Hospital referrals may also be made before 9 weeks, if clinically indicated. All abortion care is provided free of charge.

The emphasis on EMA enabled abortion services to be established quickly across a wide range of geographic locations; however, a drawback of this approach is the resulting limited availability of surgical abortion, with most patients not being offered choice of method.

In April 2020, changes to service provision were introduced in response to the COVID‐19 public health emergency, permitting the delivery of early abortion care by remote consultation (phone or video conferencing). This service delivery modality is commonly referred to as telemedicine (TM) abortion. Significantly, policymakers determined that TM abortion could be introduced without amending the abortion law.

## 2 TELEMEDICINE ABORTION

Telemedicine is broadly described by the World Health Organization as “The delivery of health care services, where distance is a critical factor, by all health care professionals using information and communication technologies…”.^3^

TM abortion services have existed both within and outside formal health systems for more than a decade in some countries. Online telemedicine services—most notably Women on Web, and Women Help Women—provide access to medical abortion in settings where abortion is legally restricted.^4^ In other locations, such as some US states and parts of Australia, TM abortion services have been established within formal health systems to improve access to abortion, particularly to reduce geographical access barriers.^5^, ^6^, ^7^ A systematic review by Endler et al.,^8^ which incorporated studies in both environments, found that TM abortion is highly acceptable to service users and service providers, with similar success rates and safety outcomes to in‐person care. Additionally, a recent major cohort study of over 50 000 women in England concluded that TM abortion is effective, safe, acceptable, and improves access to care.^9^

TM abortion services operating within formal health systems tend to require women to attend a local health facility to obtain screening tests, and frequently include the provision of ultrasound. This contrasts with the TM model as pioneered by Women on Web. Developed for use in settings where safe abortion is inaccessible, this model provides evidence for the safety of providing medical abortion without in‐person contact.^4^

The role of in‐person screening tests pre‐abortion is a notable distinction between the provision of TM abortion within formal health systems prior to and during the COVID‐19 global pandemic. The introduction of TM in Ireland was necessitated by an unprecedented public health emergency which required a substantial reduction in face‐to‐face contacts to protect the safety and well‐being both of patients and healthcare providers. In contrast to pre‐existing TM models, Ireland's revised Model of Care for abortion services during the COVID‐19 pandemic, detailed below, requires face‐to‐face visits to health facilities only when clinically indicated.

## 3 ONSET OF THE COVID‐19 PANDEMIC

The first case of coronavirus was confirmed in Ireland at the end of February 2020. Amid growing concern about its potential impact, the government and Health Service Executive (HSE) issued public health guidance to help reduce transmission. Two of the key public health messages from the HSE at this time were to reduce social interactions and keep a 2 m distance from others. At the onset of the pandemic therefore, Ireland's model of abortion care requiring two in‐person visits was located outside of this public health guidance, doubling the risk of COVID‐19 exposure both for service users and staff.

It quickly became clear that coronavirus would have significant implications for the delivery of healthcare services. The IFPA began revising its operational protocols to reduce in‐person contact, meet physical distancing requirements, incorporate personal protective equipment (PPE) usage and additional hygiene measures and, as far as possible, shift the delivery of its sexual and reproductive health services from in‐person to remote consultations.

In this context, immediate concerns were identified with respect to abortion care because it is subjected to a higher (and unwarranted) degree of legislative and regulatory oversight than other areas of sexual and reproductive healthcare. In order to access abortion in early pregnancy, the law stipulates that a pregnant woman must be examined by a medical practitioner and a mandatory waiting period of 3 days must elapse between the initial consultation and the provision of treatment.^2^ This two‐visit model is contrary to best international practice as set out by standard‐setting bodies such as the World Health Organization, which considers mandatory waiting periods to be access barriers, and the UK Royal College of Obstetricians and Gynaecologists, which recommends that abortion care be provided as soon as possible, ideally on the same day as the initial assessment.^10^, ^11^ These requirements were interpreted by the HSE in its 2019 Model of Care for termination of pregnancy services to mean two face‐to‐face consultations with a treating physician separated by at least 3 days.^12^ Consequently, this framework would need to be amended to enable the delivery of abortion care via TM.

The IFPA was concerned that some individuals would be unable to access abortion care during the pandemic unless it was provided through remote consultation. This included those in self‐isolation due to COVID‐19 symptoms or because they were at heightened risk, those with COVID‐19, and those who would not be able to attend in‐person consultations because they no longer had access to childcare facilities or schooling as a result of national lockdown measures.

On March 20, 2020, the IFPA wrote to the Minister for Health to highlight concerns regarding the challenge facing healthcare providers to observe public health guidance relating to COVID‐19 in order to protect the health and safety of clients and staff, whilst also meeting the requirements of the Model of Care and the legislation governing abortion provision. The organization proposed the following measures to enable abortion care to be delivered safely for the duration of the pandemic: (1) shifting the first consultation from in‐person to telephone; (2) identifying clients whose care could be provided in full by phone; and (3) identifying clients who may need in‐person consultations.

The IFPA Advocacy and Communications team consulted with legal experts and engaged with parliamentarians about potential amendments to the emergency COVID‐19 legislation that was making its way through the Houses of the Oireachtas (Irish parliament). These amendments sought to support the continued provision of abortion during the pandemic through: (1) giving providers discretion in determining when face‐to‐face or remote consultation is indicated; (2) giving providers discretion to waive the mandatory waiting period on public health grounds; and (3) increasing task‐sharing, by permitting nurses and midwives to carry out TM abortion consultations.^13^

In a significant development, the Minister for Health told Dáil Eireann (lower house) on March 26, 2020 that remote consultation for abortion care could be permitted without legislative amendment.^14^ In his statement, the Minister argued that the language of “having examined” in section 12 of the 2018 Act—previously interpreted to require an in‐person visit—“does not prescribe the actions or clinical aspects of the medical practitioner's examination of the woman”. Therefore, he stated, the Act does not exclude the possibility of conducting abortion consultations by telephone or video conferencing. Consequently, instead of amending the 2018 Act, telemedicine would be permitted through the issuance by the HSE of a revised Model of Care for the duration of the public health emergency, specifying that abortion consultations may take place by phone or video conferencing.

There are some further points of note in this statement regarding the framing of abortion in public discourse. It is striking that the Minister framed his contribution around the principle of access, opening with the assertion that the aim was to ensure continued access to abortion during the COVID‐19 pandemic. With respect to the pre‐pandemic in‐person visit requirement, he stated that termination of pregnancy “is and should be no different from any other health service in this way”. This is an encouraging sign of the process of normalization of abortion. However, it remains the case that government policy does exceptionalize abortion by regulating it in a legal framework that includes criminal provisions.

## 4 IMPLEMENTATION OF THE REVISED MODEL OF CARE: EXPERIENCE OF THE IFPA

When the revised Model of Care was published by the HSE on April 6, 2020, it specified that face‐to‐face consultations for abortion care should take place only when clinically necessary and such consultations should be kept to a minimum during the COVID‐19 pandemic.^15^ After the second consultation, the patient or a nominated individual can collect the abortion medications from the provider. Collection by courier is also permitted. The document further states that these are temporary provisions which will apply for the duration of the public health emergency.

The IFPA Medical Director began developing a detailed implementation plan for the organization's abortion service. The outcome was a comprehensive, interdisciplinary care pathway in which patients would have two phone consultations with a doctor before arranging for collection of their Home Care Pack from the clinic, which contained the abortion medications. The decision to deliver care by phone enabled IFPA staff to adapt to the new pathway quickly, using pre‐existing clinic infrastructure with which they were familiar rather than having to navigate a new form of technology, such as a videoconferencing platform, which would have required additional training and resourcing. The use of phone consultation also meant that people would be able to access care without the need for digital literacy or good internet access. When indicated, face‐to‐face counselling sessions and medical consultations were facilitated.

To mitigate concerns that quality of care would be compromised by the inability to see patients in‐person, a range of additional supports was built into the care pathway. This included the development of a Step‐by‐Step guide to using the Home Care Pack and a series of videos explaining how the new abortion care pathway works. These materials were a collaborative effort by clinical, counselling, and communications staff. A translation function was also added to the IFPA website to enable patients to read the information in their own language.

For the videos, staff developed a script in which the communication was clear and careful, with significant attention given to ensuring patients felt supported throughout the new care pathway. Due to physical distancing requirements, individual staff members undertook to film themselves in their homes using mobile phones, with the assistance of family or friends. A freelance videographer provided advice and support via videoconference and edited the final videos, which were then uploaded to the IFPA’s website and YouTube channel and shared on social media. Subtitles were included to improve accessibility and to enable patients to watch them discreetly.

The new care pathway effectively utilized the IFPA’s experienced specialist pregnancy counselling team who developed a three‐pronged approach, summarized by the Head of Counselling as “Information—Implications—Therapeutic” work. When people contacted the service for abortion care, reception staff took their details, booked them in for two phone consultations with a doctor, and offered them an appointment with a member of the counselling team. The counsellor then contacted the client via telephone or videoconferencing to take them through a COVID‐19 triage process, gave them practical information about what to expect during their consultations and treatment, and provided a safe space to discuss any worries or concerns they might have. They discussed the client's individual circumstances and any additional supports they might need. This provided an opportunity to identify particularly difficult circumstances or medical conditions, which could then—with the client's consent—be relayed to the doctor in advance of the first phone consultation. At this stage, an information pack was emailed to the client, containing links to the video clips, Step‐by‐Step guide, and information sheets in different languages. Counsellors also offered clients the option of a post‐abortion check‐in call. Approximately 2 weeks after taking their abortion medications, patients received a call from an IFPA nurse to discuss the result of their low‐sensitivity pregnancy test and any concerns they may have.

To assess the acceptability of this change in service delivery, patients were invited to complete an anonymous online survey after they had concluded their care with the IFPA. The survey sought feedback on service users’ interactions with different staff members, the information they received, and the perceived advantages and disadvantages of accessing abortion care by phone consultation. Those who responded were very satisfied with the overall service and the level of information provided. They identified several advantages to accessing care by phone. Most commonly, it meant they did not have to take time off work or education, it reduced their risk of COVID‐19 exposure, and it was more convenient. Some felt it gave them more privacy, enabled them to access care sooner, and meant they did not have to arrange childcare or other care or incur transport costs. Few disadvantages were identified, though two respondents felt it was hard to take in all the information and one stated that they would prefer to be able to see the person speaking to them. As the number of respondents was small, this feedback cannot be considered representative of everyone who accessed the IFPA’s service. However, the feedback is consistent with larger evaluations of similar services, such as that undertaken by BPAS in relation to its Pills by Post service.^16^

## 5 IMPLICATIONS FOR LAW, POLICY, AND SERVICE DELIVERY

Globally, there has been a wide range of legal and policy responses to the delivery of abortion care during the COVID‐19 pandemic.^17^ In Ireland, the public health emergency precipitated a rapid development in abortion provision that was not anticipated by politicians, policymakers, or service providers at the time of abortion law reform only 18 months earlier. The country has moved from being an outlier in Europe due to its extremely restrictive abortion laws, to becoming one of only a handful of European countries to introduce progressive reforms during the pandemic by permitting telemedicine abortion.^18^ This approach by the Irish government and health service recognized abortion as essential, time‐sensitive healthcare and prioritized people's right to access the service.

Furthermore, whereas the introduction of TM abortion in England, Scotland, and Wales involved legal change (with sunset clauses included in England and Wales), the Irish government determined that remote consultation could be introduced without amending the 2018 abortion law.^19^ The introduction of TM abortion without legislative change was significant and demonstrates the potential for the regulatory framework to expand in ways that are patient‐centered and rights‐based. This allows the service to be more flexible and responsive to the needs of service users. It is possible, therefore, that further evidence‐based modifications to abortion care could be introduced without the need for legislative amendment.

While the revised Model of Care specifies that it will apply “for the duration of the COVID‐19 public health emergency”, the new Minister for Health has clarified in response to parliamentary questions that the model will be reviewed once the pandemic is declared over.^15^, ^20^ This opens up the possibility of retention of remote consultation beyond the COVID‐19 pandemic as part of a blended model of abortion care. The provision of TM abortion is supported by a strong international evidence base which demonstrates that it has similar outcomes to in‐person care.^8^, ^9^ The permanent adoption of TM abortion has also been endorsed by the International Federation of Gynecology and Obstetrics (FIGO), who describe it as a safe and private method that can improve abortion access and reduce exposure to stigma.^21^ Its retention within Ireland's Model of Care would demonstrate respect for women's determination of their own healthcare needs, in somewhat the same way that pregnancy is mostly dated by women's own determination of LMP, with ultrasound scanning used only if clinically required.

Furthermore, maintaining remote consultation as an option within the abortion care pathway could potentially improve access for a range of individuals, such as those living in rural areas, disabled people, and people with care responsibilities for whom in‐person appointments may be logistically challenging. It would give patients more choice in service delivery modality, enabling them to access care in a manner consistent with their needs and preferences. Such an approach would give healthcare providers more flexibility to respond to the differing circumstances of each patient and could contribute to the reduction of geographical access barriers. There are no official published data on the geographical spread of abortion providers in Ireland, although it is known that only half of the country's maternity units (10/19) provide the full range of abortion services, and there is one county with no community‐level provider. Women on Web report that some individuals continued to seek abortion by online telemedicine in the first 3 months following legalization.^22^ Furthermore, the 2019 abortion statistics for England and Wales show that 375 women gave Irish addresses when accessing abortion services, including women who were legally eligible for care in Ireland.^23^ Travelling abroad for abortion care has become even more complex and burdensome in the context of the global pandemic.^24^

The decision by the Irish government to take action in order to maintain abortion access in the context of a public health emergency which placed immense pressure on health services is a positive development and lays the foundation for a legislative review process (due to commence in 2021) that is patient‐centered and focused on enhancing access to care. However, telemedicine alone will not address all access barriers—the Irish abortion law still contains provisions that delay access to care and, in some instances, exclude individuals from accessing care entirely.^25^ This includes a 12‐week gestational limit and mandatory waiting period as well as extremely narrow grounds for abortion access post‐12 weeks. Abortion care is only accessible after this gestational cut‐off when there is a risk to the life or of serious harm to the health of the pregnant woman, or when a condition is present which is likely to lead to the death of the fetus (commonly referred to as a ‘fatal fetal anomaly’). Neither ground meets international human rights standards.^26^ In the first year of service provision, out of a total of 6666 abortions provided under the new law, only 21 women accessed abortion under the risk to life/health ground.^27^ Medical practitioners providing abortion in cases of fatal fetal anomaly have highlighted the challenges of working under ‘ambiguous’ and ‘restrictive’ legislation that contains the threat of criminal sanctions.^28^ Moreover, further work is needed to underpin the legislation with ethical guidance for healthcare providers regarding women's agency, health and rights.^29^ The upcoming legislative review provides a critical opportunity to address these shortcomings, to learn from service users and providers who have accessed and provided care under this framework for the past 2 years and to draw on international evidence concerning best practice and human rights standards.

## 6 CONCLUSION

International evidence indicates that TM abortion is safe, effective, and acceptable to service users and may contribute to the reduction of access barriers, particularly geographical access barriers. In the Irish context, the experience of the IFPA as a community provider of EMA, along with feedback received from service users, indicates that this mode of service delivery works well both for people seeking access to abortion and healthcare providers. Its retention as part of a blended approach to abortion provision would expand service user choices and support their reproductive autonomy. It is therefore welcome that the government has committed to reviewing the remote consultation model, rather than withdrawing this service delivery modality in its entirety, once the COVID‐19 public health emergency has concluded. The introduction of remote consultation has contributed to the recognition of abortion by the State as essential and time‐sensitive healthcare. We suggest that the actions taken by the government and health service during the pandemic constitute a patient‐centered, rights‐based approach to abortion care, and lay the foundation for a legislative review process that is focused on the needs of service users, with the aim of enhancing access to abortion care in law and practice for all who need it.

## CONFLICTS OF INTEREST

The authors have no conflicts of interest.

## AUTHOR CONTRIBUTIONS

AS devised the article in consultation with MT and CC and developed the first draft. Written feedback from MT and CC was incorporated to produce a further draft. This was shared with CH and RV who provided input based on their respective clinical and counselling expertise. All authors contributed to and approved of the final version of the manuscript.
